# Vascular Disease Patient Information Page: Livedoid vasculopathy

**DOI:** 10.1177/1358863X221128620

**Published:** 2022-10-26

**Authors:** Harish Eswaran, Priyanka Vedak, Paul Googe, Stephan Moll

**Affiliations:** 1 Department of Medicine, University of North Carolina School of Medicine, Chapel Hill, NC, USA; 2 Department of Dermatology, University of North Carolina School of Medicine, Chapel Hill, NC, USA; 3 Dermatopathology Laboratory, Department of Dermatology, University of North Carolina School of Medicine, Chapel Hill, NC, USA; 4 Department of Medicine, Division of Hematology, University of North Carolina School of Medicine, Chapel Hill, NC, USA

**Keywords:** livedoid vasculopathy, skin, thrombotic vasculopathy, wound/ulcer

## What is livedoid vasculopathy (LV)?

Livedoid vasculopathy, or LV, is a chronic skin condition characterized by small, painful sores that come and go over the legs and feet. ‘Livedoid’ refers to the bluish skin discoloration that often accompanies these sores. ‘Vasculopathy’ means a disease of the blood vessels. LV has been called different names such as livedoid vasculitis and *atrophie blanche*. LV is a rare disease, occurring in less than one in 100,000 people per year.^1^

## What are the symptoms of LV?

**Figure 1.**
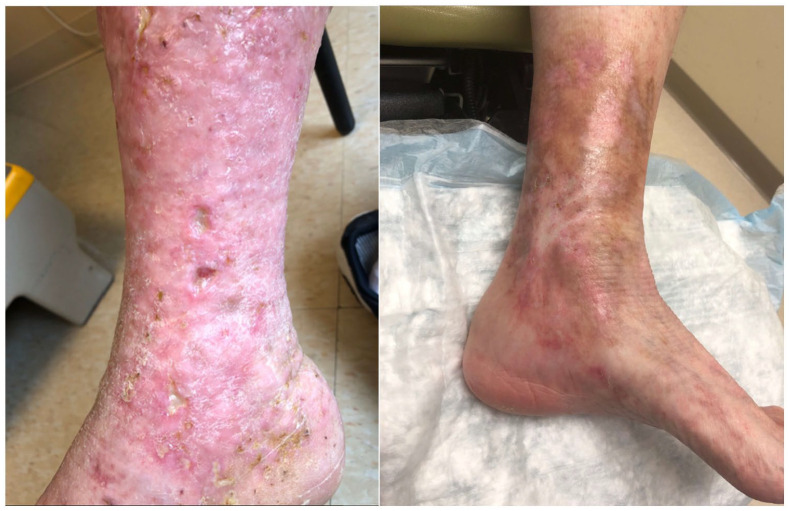
Examples of livedoid vasculopathy. **Left**: Small, deep sores on a background of red, discolored skin. **Right**: Healed ulcers with white, star-like scars referred to as atrophie blanche.

Skin changes in LV occur in three stages (Figure 1):

- Reddish-purple patches appear on the skin first.
- Small, deep, and painful open sores—called ulcers—form within these patches.
- Ulcers take months to heal, forming white scars known as atrophie blanche.

Multiple sores about the size of a pencil eraser may exist at the same time over both legs in different stages of healing. It is also common for patients to experience flares of painful ulcers in between periods with minimal symptoms. The triggers for these flares are unclear and often none can be identified. About a third of patients notice a relationship between their symptoms and warm weather.^2^

## What causes LV?

**Figure 2.**
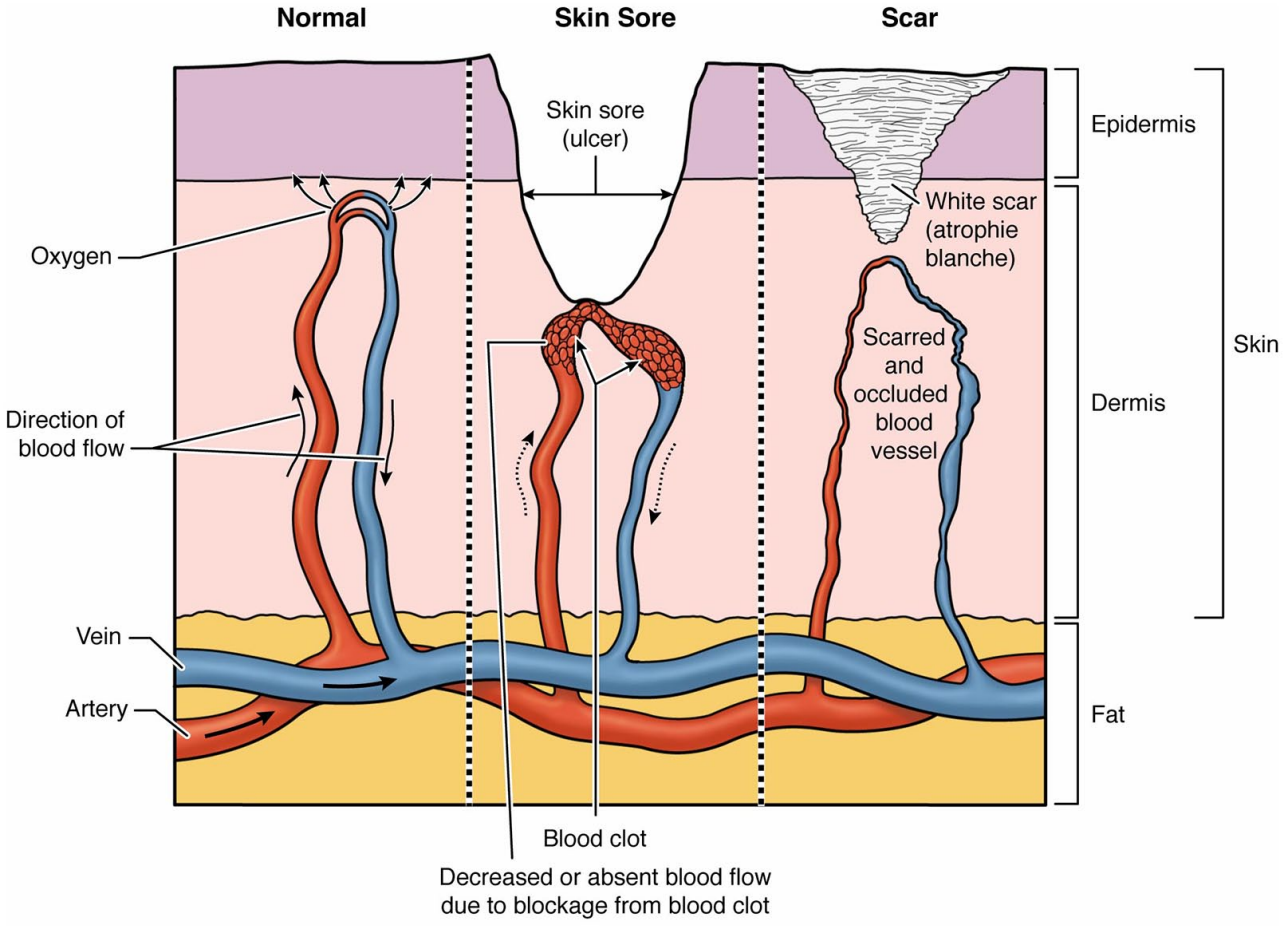
Livedoid vasculopathy under the microscope. Clots form in tiny blood vessels in the skin, starving skin tissue and causing ulcers to form. Ulcers eventually heal as white, star-like scars.

LV is caused by blood clots in the tiny blood vessels in the skin (Figure 2). These blood clots can only be seen under the microscope after a skin biopsy. They are much smaller than the ones that cause clots in the large veins of the leg known as deep vein thrombosis (DVT). Blood clots in LV cut off the flow of oxygen to the skin, causing the skin tissue to die and form ulcers.

The exact cause of these small clots is unknown. Most of the time LV occurs in the absence of other medical conditions.^3^ However, it may be associated with diseases that predispose patients to form blood clots—known as clotting disorders, thrombophilias or hypercoagulable states—such as antiphospholipid syndrome.^4^ LV may also be associated with autoimmune diseases (such as lupus or rheumatoid arthritis) or with chronic venous insufficiency, a condition that causes leg swelling and varicose veins. Genetics may play a small role, but LV does not typically run in families and it is not transmissible from person to person.^5^

## How is LV diagnosed?

Doctors may suspect LV after talking with patients about their symptoms and examining their legs; however, a biopsy of the skin is required to diagnose LV. This procedure is usually performed by a dermatologist, a specialist in skin diseases. Typical LV biopsies show clots within the small blood vessels of the skin with very little surrounding inflammation.^1^ It may take more than one biopsy to confirm the diagnosis because LV can be patchy and affected segments of blood vessels may be missed in a single biopsy.

## What other conditions may cause symptoms similar to LV?

Many conditions can cause skin ulcers of the legs and are more common than LV. These include diabetes, chronic venous insufficiency, and peripheral artery disease. It is important to look for these before making a diagnosis of LV. Doctors may order lab tests for diabetes, autoimmune disease, thrombophilia, cancer, or infections. They may also order a vascular ultrasound of the legs to look for problems with blood flow. The skin biopsy can help to rule out vasculitis, a different condition caused by inflammation and destruction of blood vessels. LV should also not be confused with livedo reticularis, a red or blue, net-like skin discoloration that does not cause clinical problems like sores or pain.

## How is LV treated?

Treatment of LV consists of pain management, wound care, medicines that address the blood clots that cause the ulcers, and medicines that suppress the immune system (Table 1).^6^^7^ Treatment often requires collaboration among different medical specialties, such as dermatologists, wound care specialists, hematologists, rheumatologists, vascular medicine specialists, and/or vascular surgeons.

**Table 1.** Treatments for livedoid vasculopathy.

Treatment class	Examples
Wound care	Surgical debridement
	Hyperbaric oxygen
	Compression stockings
	Ultraviolet light
Blood thinners	Anticoagulants
	Antiplatelet agents
	Thrombolytics (clot busters)^a^
Immune suppression/modulation	Steroids
	Immune modulators
	Intravenous immunoglobulin (IVIG)^a^

a Generally administered in the hospital setting.

Wound care for LV includes regular wound cleaning, surgical removal of broken-down skin tissue (debridement), hyperbaric oxygen, compression therapy, and ultraviolet (UV) light. These treatments are often administered in a wound care clinic. Hyperbaric oxygen is delivered in a sealed chamber in which oxygen levels are maximized to revitalize skin tissue. Compression stockings can be worn at home and help with ulcer healing by preventing swelling of the legs that may worsen skin breakdown.^8^ UV light may prevent excessive inflammation around the wounds. Antibiotics may be needed if ulcers are infected.

Blood clots in the skin in LV are made up of clotting proteins as well as a type of blood cell called platelets. Two types of medications can be used to prevent blood clots from forming or growing bigger: anticoagulants and antiplatelet agents. These drugs are collectively referred to as blood thinners. Anticoagulants prevent clotting proteins from forming clots. The most commonly prescribed treatments for LV are direct oral anticoagulants (DOACs).^6^ Examples of DOACs include apixaban (Eliquis), dabigatran (Pradaxa), and rivaroxaban (Xarelto). DOACs are generally taken once or twice a day and do not require blood level monitoring. For more information, please see the patient information page on DOACs.^9^ Two other types of anticoagulant drugs are the oral warfarin (Jantoven) and injectable low-molecular-weight heparins (LMWH), such as enoxaparin (Lovenox), dalteparin (Fragmin), and tinzaparin (Innohep). Antiplatelets block platelets from sticking together. These may be prescribed in addition to or in place of anticoagulants for LV. Examples of antiplatelets include aspirin, clopidogrel (Plavix), or pentoxifylline (Trental). It has not been studied whether one type of blood thinner is more effective than another for the treatment of LV.

Many other treatments have been tried in LV with variable results. Other medications are sometimes found to be helpful, particularly if LV is associated with another disease. People who have an autoimmune disease, such as lupus or rheumatoid arthritis, in addition to LV may benefit from medications that suppress the immune system, such as steroids. Sometimes, LV is severe enough to require hospitalization. Inpatient treatments include tissue plasminogen activator (tPA), a strong medication that breaks up blood clots, and immune-modulating therapies such as intravenous immunoglobulin (IVIG) and rituximab. More research is needed on whether any one treatment is better than another, and which patients might benefit from specific treatment types.

## What is the prognosis for LV?

LV usually begins in early adulthood, and painful ulcers can come and go throughout one’s life. Pain can be severe and interfere with work and recreation. However, patients can also experience months or even years free of symptoms. Based on current evidence, LV does not affect blood vessels in other parts of the body or cause damage to other organs like the heart, brain, or kidneys, and is not life-threatening.

## Can LV be prevented?

No one knows exactly what causes LV or how to prevent it from occurring; however, the treatments described above can help ulcers go away. Patients with LV may need to take blood thinners for life to make it less likely that new ulcers will form.

## Summary

Livedoid vasculopathy is a rare condition characterized by small ulcers, reddish-purple skin discoloration, and scars (atrophie blanche) on the lower legs that come and go without a clear trigger. LV can occur in previously healthy people, or it can be associated with diseases that predispose patients to blood clots. It may also be associated with autoimmune diseases such as lupus or rheumatoid arthritis, or with chronic venous insufficiency. Diagnosis of LV requires a skin biopsy that shows clots in the small blood vessels of the skin with very little inflammation. Repeat skin biopsies may be needed, as affected vessels can be missed on a single biopsy. Treatment options include diligent wound care, antibiotics if the ulcers are infected, blood thinners (anticoagulation and/or antiplatelet agents), and immune-modulating medications.

The ‘Vascular Disease Patient Information Page’ is a regular feature of *Vascular Medicine*. All articles in the collection are available for free online at http://journals.sagepub.com/vmjpatientpage. The Vascular Disease Patient Information Page is provided for educational purposes only and is not a substitute for medical advice.
